# Impact of a lay-led home-based intervention programme on quality of life in community-dwelling pre-frail and frail older adults: a randomized controlled trial

**DOI:** 10.1186/s12877-017-0548-7

**Published:** 2017-07-19

**Authors:** A. Kapan, E. Winzer, S. Haider, S. Titze, K. Schindler, C. Lackinger, T. E. Dorner

**Affiliations:** 1 Centre for Public Health, Department of Social and Preventive Medicine, Medical University of Vienna, Kinderspitalgasse 15/1, 1090 Vienna, Austria; 2 Institute of Sport Science, University of Graz, Graz, Austria; 3 Division of Endocrinology and Metabolism, Department of Medicine, Medical University of Vienna, Vienna, Austria; 4 Department of Health Promotion and Prevention, Sportunion Austria, Vienna, Austria

**Keywords:** Frailty, Quality of life, Volunteer, Home-based programme

## Abstract

**Background:**

Due to the demographic ageing process and the increasing number of pre-frail and frail individuals, new lifestyle interventions to enhance the quality of life (QoL) in community-dwelling older adults are necessary. Therefore, we performed a randomised controlled trial (RCT) to compare effects of a lay-led home-based physical and nutritional intervention programme with social support alone on different QoL domains in community-dwelling pre-frail and frail older adults.

**Methods:**

In this analysis within a RCT (12 weeks), lay volunteers visited one-on-one pre-frail or frail older adults at home twice a week. Participants in the physical training and nutritional intervention (PTN) group performed six strength exercises and discussed main nutritional issues during each visit. The social support (SOSU) group received home visits twice a week for social exchanges. The QoL was assessed with the WHOQOL-BREF and the WHOQOL-OLD instruments. Analyses of covariance (ANCOVA) were used to examine differences between groups with baseline values as the covariate. Changes within groups were assessed with paired t-tests.

**Results:**

Eighty participants (*n* = 39 in the PTN group and *n* = 41 in the SOSU group) were included. No significant differences were found between the two groups except in past, present and future activities domain [β = 3.66 (95% confidence interval 0.13 to 7.18)] in favour of the PTN group. However, there was some evidence of greater within group improvements in the PTN group particularly in overall QoL, social relations and social participation. In the SOSU group, no significant effect was observed in any QoL domain.

**Conclusion:**

A combination of a home-based physical and nutritional intervention was not more effective compared to social support alone, on QoL in community-dwelling pre-frail and frail older adults. However, the small but significant improvement within the PTN group suggests that a home-based physical and nutritional intervention delivered by volunteers may influence the QoL in a positive way.

**Trial registration:**

The study protocol was registered on 6 November 2013 at ClinicalTrials.gov (identifier: NCT01991639).

## Background

Frailty is a serious emergent health-related problem in older adults [1]. Several studies have shown that functional disabilities and limitations on independency, caused by the frailty syndrome are associated with poor quality of life (QoL) [2–4], while others studies have shown that low QoL among older adults is linked with various negative health outcomes, including falls and nursing home placement [5]. In this context, numerous studies reflect that a broad majority of older people want to remain in their own homes as long as possible. Nevertheless, aging in place can become difficult if functional ability and the related meaningful activities (e.g. take care of the household, running daily errands) are no longer possible to be independently performed [6, 7]. Independence and functional ability thus plays an important role for maintaining physical and psychological health in frail older adults [5].

However, it is not only the objective changes in health and social structures that are important for the living situation in old age, but also the number of social contacts as well as the quality of social relations, which are associated with morbidity and mortality [8]. The higher the age, the smaller will the social network be. Especially under conditions of frailty, this has unfavourable effects on the QoL [9]. Consequently, older adults’ QoL can be improved and maintained at a high level, even with poor physical health, as long as they are satisfied with other dimensions of QoL, such as mental health, social and environmental relationships, and socioeconomic status [10]. Hence, a broad-ranging evaluation of QoL has become an essential component of many clinical studies across a wide range of patient populations [11].

The main objective of exercises for older adults is to increase their physical capacity to perform daily activities, such as getting out of a chair or climbing stairs and with it maintaining functional independence and QoL [12, 13]. The loss of strength and impaired static and dynamic postural control lead to increased frequency of falls, often with serious consequences with the loss of autonomy and decreased QoL [14]. The effects which can be achieved through resistance training, demonstrate the need for muscle training in old age [15]. Apart from the degree of frailty, the format and intensity of the exercise intervention, evidence indicates that physical activity and a balanced, healthy diet can improve and maintain the QoL in older adults [16–19].

Due to the demographic development, were there is a growing percentage of homebound frail old people [20] and apart from the basic need for at home care, the provision of exercise intervention and social support for frail older people has become a key concern of Austria’s social policy. Additionally, according to the Austrian Volunteer Report [21] the largest area of formal voluntary work is in the field of sports and exercise. There is already good evidence that volunteering can yield double benefits, for the recipient and the volunteer themselves, in terms of improved self-esteem, well-being and social engagement [22–24]. Our research team recently demonstrated that a lay-led home-based physical activity and nutritional intervention programme can increase handgrip strength, physical performance, nutritional and frailty status [25, 26]**.** To further explore whether such an approach can influence QoL, we compared the effects of a lay-led home-based physical and nutritional intervention programme with social support alone on various QoL dimensions in community-dwelling pre-frail and frail older adults. In addition we also wanted to know which factors were associated with improvements in several QoL domains.

## Methods

### Study design

This study was conducted by the Centre for Public Health at the Department of Social and Preventive Medicine, Medical University Vienna, Austria, from September 2013 to June 2015. The study protocol [27] was registered at ClinicalTrials.gov (identifier: NCT01991639) and was approved by the local ethics committee of the Medical University of Vienna (EK No. 627/2011). All study participants signed written informed consent for participation. The study methods were in accordance with the CONSORT guidelines for reporting randomised trials [28]. Primary aim of the study was to evaluate changes in handgrip strength in a group with exercise and nutrition intervention compared to a group with social support alone, with results published elsewhere [25]. Quality of life was prospectively defined as secondary outcome in this trial, and the results are presented in this paper.

### Sample size

Details of the sample size calculation, based on the primary outcome of the study, have been previously published [27]. In terms of power, we assumed there would be a dropout rate of 20% (including loss to follow-up). In order to observe a clinically important difference of 2 kg (standard deviation of 3) in handgrip strength between the physical training and nutritional intervention (PTN) and social support (SOSU) groups at 12 weeks, we estimated that a total sample size of 80 persons (40 in each group) was required for 80% statistical power. For this purpose, sex-specific cut-off values (male: 22 kg; female: 15 kg) based on the median values of our pre-study were used.

### Randomisation

Participants meeting the inclusion criteria were randomly assigned to the PTN group or the SOSU group. Based on the results of the pre-study [29], the randomisation was stratified by using sex-specific handgrip strength thresholds (males <22 kg and females <15 kg) and was centralized using the “Randomiser for Clinical Trials 1.8.1” [30].

### Participants

#### Pre-frail and frail older adults

The eligibility criteria for recruiting were community-dwelling pre-frail or frail persons, according to the Frailty Instrument for Primary Care of the Survey of Health, Ageing and Retirement in Europe (SHARE-FI) [31]. The SHARE-FI instrument consists of five items, i.e., “exhaustion”, “loss of appetite”, “weakness”, “slowness” and “low physical activity”. The ratings of the five items are summarized to form a discrete factor score (DFS). Hence, patients can be classified as “frail”, “pre-frail” and “non-frail”. Additionally, malnourished persons (or persons at risk of malnutrition) according to the Mini Nutritional Assessment Short-Form (MNA-SF ≤11 points) [32] were included. Further inclusion criteria were that the participants had to be older than 65 years, live in their own homes and had to be able to walk (with or without a walking aid).

Individuals with the following diseases were excluded: chronic obstructive pulmonary disease stage III or IV, impaired cognitive function of ≤17 points according to the Mini Mental State Examination (MMSE) [33], planned admission to nursing home, cancer with on-going chemo- or radiotherapy, chronic kidney insufficiency with protein restriction or dialysis, insulin-treated diabetes mellitus or a higher-grade nursing level (more than 180 h of care per month is necessary).

### Lay volunteers (buddies)

In cooperation with “Wiener Hilfswerk”, a non-governmental provider of social care services, the lay volunteers (aka “buddies”) underwent a structured interview with a psychologist in terms of personal motivation and their intention for participation. Further inclusion criteria were that the buddies had to be older than 50 years and willing to conduct two home visits weekly. Before the buddies were included in the study, they underwent a four-day training course. In the course, the buddies learned to perform the different exercises. Frailty, important nutritional messages, social interaction and safety in the exercises were also discussed and practised. The main elements of this programme were interactive role play and simulation exercises in order to ensure the lay-person’s confidence, underpinning the practical workshops. Moreover, a telephone hotline to health professionals, including a physiotherapist and a dietician, was established in order to provide an additional level of safety for the buddies.

### Physical training and nutritional intervention (PTN) group

Buddies visited the pre-frail or frail participants for 12 weeks, twice a week, for approximately 1 h. The prescribed structured intervention programme was based on the recommendations of the American College of Sports Medicine and the American Heart Association [34] and was structured as follows: the training unit lasted about 30 min and included a 5-min warm-up (mobilisation) and six strength exercises, which were performed in two sets, with 12–15 repetitions, until muscular exhaustion, on the principle of progressive increasing exertion. The strength training included the following exercises: mini squats in front of a chair, “beetles” exercise for the abdominal muscles, hip extension in standing position, reverse butterfly, chest press and shoulder press against elastic resistance. The exercises were designed to stimulate all major muscle groups. In addition, the importance of health-enhancing physical activity was discussed with the participants. A total of eight nutritional messages (such as fluid intake, animal and plant protein, energy intake), were discussed during each home visit. As an assistive device for the nutritional topics, buddies and the pre-frail or frail individuals were provided with a handbook covering all eight nutritional themes. In addition, participants were provided with the “Healthy for Life Plate”, which is a modification of the “Healthy Eating Plate” [35], to show the difference between recommended and actual food intake.

### Social support (SOSU) group

The buddies visited the pre-frail or frail participants twice a week for social meetings only. The SOSU group served as an active control group. In addition to the social contact, ideas for cognitive exercises (e.g. memory card, card games or they describe a random objective) were provided. However, the participants were allowed to spend their time as they wanted to (e.g. talk about their life experiences).

### Measures

All measurements and questionnaires were completed in the home environment of the pre-frail or frail older adults by the study team. All measurements and questionnaires were completed before the intervention session (baseline) and after 12 weeks. For the sake of clarity, the following nutritional and physical covariates as primary outcome, which were published previously [25, 26], were added to explain the QoL and its changes. The adherence rate was monitored using diaries kept by the buddies. Any reports of adverse events associated with the intervention programme were also recorded.

There are two questionnaires frequently used to evaluate QoL; the WHOQOL-BREF with four domains (physical, psychological, social and environmental aspects of QoL) and the WHOQOL-OLD, an add-on module, which is used in combination with the WHOQOL-BREF, with six domains (sensory abilities; autonomy; past, present and future activities; social participation; death and dying; and intimacy) [36, 37]. Thus, these domains have been identified as highly relevant for the general elderly population and should be taken into account when evaluating their QoL [38]. The WHOQOL-BREF is a 26-item short-form rated on a five-point Likert scale, which is calculated according to the standard protocol by multiplying the mean of all the items included within the domain by a factor of four. In this study, in contrast to the original scoring instructions of the World Health Organization, the social relationships domain was calculated using two instead of three items, because less than 10% of the participants replied to the question “How satisfied are you with your sex life?” The WHOQOL-BREF has a good test reliability, i.e., a Cronbach’s alpha score of 0.79 [36]. In addition to the WHOQOL-BREF, the WHOQOL-OLD [37] instrument was also used, because it specifically evaluates QoL in an elderly population. It is also recommended for use in combination with the WHOQOL-BREF [39]. Each item was scored on a Likert scale ranging from 1 to 5. The sum of the domains was transformed into a 0–100 score.

The Short Physical Performance Battery (SPPB) [40] was used to assess physical performance. The SPPB consists of standing balance tests (side-by-side, semi-tandem and tandem stand), a timed 4-m walk and a timed test of five repetitions of rising from a chair and sitting down, all ranging from 0 points (lowest score) to 4 points (highest score). Finally, the physical performance score ranges from 0 (worst) to 12 (best). Daily physical activities such as leisure time, household and work-related activities were assessed using the Physical Activity Scale for the Elderly (PASE) [41]. This is a self-reported questionnaire that covers a single week. Results can range from 0 to 360, as the sum of the amount of time in each activity is multiplied by the weight of the activity. Handgrip strength was measured with a Jamar© hydraulic hand dynamometer. For each side, three maximum attempts were made and the best score was used for the analysis. Nutritional status was assessed with the Mini Nutritional Assessment Long-Form (MNA®-LF). The MNA®-LF [32] consists of 18 items concerning housing, weight loss, medicine use, BMI, dietary intake and arm and calf circumference. The maximum score is 30 points; 24–30 points indicates that the person is normal nourished; 17.5–23.5 points indicates at risk of malnutrition; and <17 points indicates malnutrition. Finally, cognition was measured using the German version of the MMSE [33].

### Statistical analyses

**Fig. 1:**
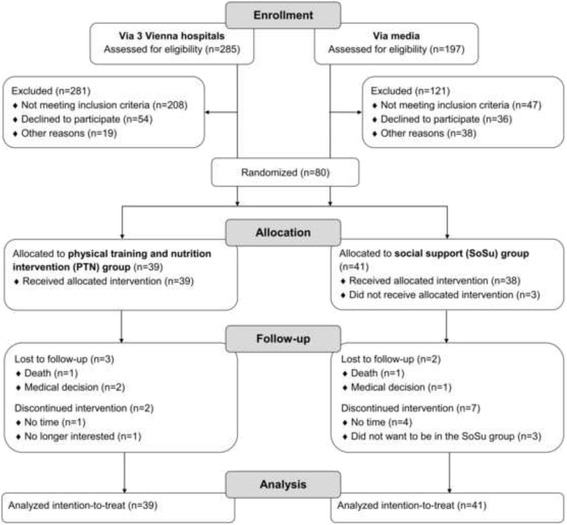
Flow of participants through the trial

To describe the sample characteristics, baseline data were compared between the PTN group and SOSU group, with the use of either unpaired t-tests (for continuous measures) or chi-square tests and F-tests (for categorical characteristics). Results are expressed as means (standard deviation) or frequencies (percentages). For all the statistical tests, an intention to treat analysis was used (all randomized patients in the groups to which they were randomly assigned were included irrespective of whether the intervention was completed or not). Missing values as shown in Fig. 1 (e.g. lost to follow-up, discontinued intervention), were imputed with the use of the last observation carried forward approach for measurements made after baseline. Thus, subjects with baseline data who were lost to follow-up were also included in the analyses. The last observation carried forward approach has been shown to be common elsewhere [42].

To assess the differences between groups with regard to the WHOQOL-OLD and WHOQOL-BREF domains, an analysis of covariance (ANCOVA) [43] was used, with the follow-up value as the dependent variable (WHOQOL-OLD and WHOQOL-BREF domains), with the group (treatment) as the fixed between-subjects factor, and baseline WHOQOL-OLD and WHOQOL-BREF scores as a covariate, additionally adjusted for age and sex. Paired t-tests were used to assess changes within groups pre- to post-intervention.

Further, several multiple linear regression analyses were used to estimate the association between changes from baseline to follow-up (post-test minus pre-test) in the nutrition and physically related parameters (MNA®-LF score, SPPB score, maximum handgrip strength, PASE score), group, sex and age with change (post-test minus pre-test) in various QoL domains. We used those QoL domains, where we found a significant improvement in the PTN group (overall QoL, social relationship, social participation, past, present and future activities) at follow-up. Thus, we undertook a stepwise multiple linear regression analysis including all independent variables (MNA®-LF score, SPPB score, maximum handgrip strength, PASE score) and group, adjusted for sex and age. A value of less than <.05 was considered statistically significant. IBM® SPSS® Statistics for Windows Version 20 software (IBM Corp., Armonk, NY, U.S.) was used for all the statistical analyses.

## Results

### Study population

As can be seen in Fig. 1, 197 older adults recruited via the media and 285 older adults recruited via three hospitals were included in this study. After exclusion of the participants not meeting the inclusion criteria, 80 participants were randomised, whereby 39 subjects were allocated to the PTN group and 41 to the SOSU group. The baseline characteristics of the included participants are shown in Table 1. The mean age of the participants was 82.6 (SD 8.1) years, and 84% were women. One-quarter of the participants were ≥90 years old and three-quarters lived alone. Between the PTN and SOSU groups, there was no significant difference at baseline in any measure.

**Table 1:** Baseline characteristics of the participants of the physical training and nutritional intervention (PTN) group and the social support (SOSU) group

Variable	PTN (*n* = 39)	SOSU (*n* = 41)	*P*-value
Sex			
Female n (%)	33 (84.6)	34 (82.9)	0.838
Age: years mean (SD)	83 (8.0)	82.5 (8.0)	0.755
65–79 n (%)	13 (33)	13 (32)	0.733
80–89 n (%)	15 (39)	19 (46)	
≥ 90 n (%)	11 (28)	9 (22)	
Living alone			
Yes n (%)	32 (82)	35 (85)	0.688
Children around			
Yes n (%)	30 (77)	34 (82)	0.502
Educational level			
Primary n *(%)*	23 (60)	18 (45)	0.410
Secondary n *(%)*	12 (30)	16 (40)	
Tertiary n *(%)*	4 (10)	7 (15)	
Frailty status			
Robust n (%)	1 (3)	0	0.523
Pre-frail n (%)	14 (36)	14 (34)	
Frail n (%)	24 (62)	27 (66)	
Cognitive function			
No cognitive impairment (%)	32 (82)	30 (73)	0.342
Mild cognitive impairment (%)	7 (18)	11 (27)	
SPPB score mean (SD)	5.2 (2.9)	4.9 (2.8)	0.639
Maximum grip strength kg mean (SD)	16.1 (7.4)	17.5 (6.9)	0.394
PASE score mean (SD)	33.8 (37.1)	31.7 (31.6)	0.820
MNA®-LF score mean (SD)	23.8 (3.4)	24.2 (3.13)	0.588
WHOQOL-BREF score mean (SD)	63.79 (15.1)	66.13 (10.1)	0.449
Overall QoL mean (SD)	40.76 (19.2)	46.83 (16.1)	0.102
Physical health mean (SD)	49.08 (18.7)	48.17 (13.3)	0.816
Psychological health mean (SD)	58.97 (18.5)	63.01 (13.3)	0.264
Social relationship mean (SD)	72.43 (23.7)	77.43 (19.8)	0.308
Environment mean (SD)	74.67 (14.3)	75.91 (10.8)	0.663
WHOQOL-OLD score mean (SD)	47.88 (11.9)	51.40 (10.1)	0.274
Sensory abilities mean (SD)	47.43 (23.6)	49.82 (21.9)	0.633
Autonomy mean (SD)	51.28 (16.4)	56.09 (13.3)	0.152
Past, present and future activities mean (SD)	49.74 (16.3)	53.78 (14.3)	0.240
Social participation mean (SD)	43.07 (15.1)	45.85 (10.7)	0.344

*Note*: Data are presented as mean (standard deviation) for continuous variables and percentages for categorical variables

*PTN* physical training and nutritional intervention group, *SOSU* social support group, primary = elementary school or no degree; secondary = secondary school; tertiary = university entrance diploma or higher degree, *SPPB* Short Physical Performance Battery, *PASE* Physical Activity Scale for the Elderly, *MMSE* Mini Mental State Examination, *MNA®-LF* Mini Nutritional Assessment Long-Form

### Drop-out rate, adherence and adverse events

According to Fig. 1, 14 persons dropped out, amounting to 18% of the baseline population. Five of the 39 participants in the PTN group (12.8%) and nine of the 41 participants (21.9%) from the SOSU group dropped out in the course of this trial. The main cause of discontinuations was that the participants in the SOSU group were disappointed not to be in the PTN group and lack of time. There were no significant differences between the PTN and SOSU group in respect to frequency (PTN group 18 ± 4.9 and SOSU group 14 ± 5.2 home visits) and duration (PTN group 94 ± 29 min and SOSU group 82 ± 31 min per home visit). One participant in the intervention group reported an adverse event (back pain) that may have been associated with the exercise program.

### Effects on outcome parameters

At the end of the 12-week intervention, a significant between-group difference (Table 2) in past, present and future activities, in favour of the PTN group, was observed. No significant differences between groups were found in all the other QoL domains. However, a significant between-group difference in the physical activity related parameters of the SPPB score and PASE score, but not in maximum handgrip strength and MNA®-LF score, was observed in the PTN group. Although significant within-group changes were found over time in overall QoL, social relations and social participation, in favour of the PTN group, no between-group changes were found regarding these outcomes. In contrast, no significant changes within the SOSU group were found in any QoL domain.

**Table 2:** Changes in different QoL dimensions and nutritional and physical parameters from baseline to 12 weeks

Variable	PTN group	SOSU group	Difference between groups	
	Mean change (95% CI)	Mean change (95% CI)	ß^a^ (95% CI)	*p*-value^b^
WHOQOL-BREF domains				
Overall QoL	5.6 (0.95 to 10.33)*	2.5 (−1.66 to 6.54)	3.16 (−2.59 to 8.91)	0.277
Physical health	3.3 (−1.33 to 7.92)	3.4 (−0.59 to 7.73)	−0.49 (−6.13 to 5.15)	0.843
Psychological health	2.9 (−0.67 to 6.52)	0.2 (−2.84 to 3.04)	2.23 (−2.20 to 6.65)	0.320
Social relationship	4.5 (0.38 to 8.59)*	1.5 (−4.34 to 7.38)	1.36 (−5.13 to 7.83)	0.678
Environment	1.4 (−2.15 to 4.87)	1.2 (−2.53 to 4.96)	−4.12 (−4.84 to 4.02)	0.854
WHOQOL-OLD domains				
Sensory abilities	4.5 (−1.36 to 10.34)	0.6 (−3.83 to 5.04)	3.12 (−3.37 to 9.57)	0.141
Autonomy	2.7 (−0.45 to 5.97)	1.5 (−2.60 to 5.61)	−0.59 (−5.13 to 3.93)	0.786
Past, present and future activities	4.7 (1.99 to 7.42)*	−0.1 (−3.23 to 2.95)	3.66 (0.13 to 7.18)	0.039
Social participation	3.8 (0.12 to 7.57)*	2.5(−1.33 to 6.46)	0.44 (−4.64 to 5.52)	0.870
Nutritional and physical parameters				
MNA®-LF Score	1.6 (0.53 to 2.59)*	0.9 (−0.27 to 2.22)	0.28 (−1.12 to 1.69)	0.689
SPPB total score	1.2 (0.35 to 2.15)*	0.5 (0.12 to 0.93)	1.01 (0.03 to 1.99)	0.044
Maximum handgrip strength	2.1 (0.77 to 3.62)*	1.0 (−0.27 to 2.25)	0.86 (−0.82 to 2.49)	0.318
PASE total score	17.1 (9.13 to 25.01)**	1.9 (−3.93 to 7.79)	15.12 (5.44 to 21.68)	< 0.001

*Note*: Mean change (95% CI) is reported for continuous variables at baseline and after 12 weeks

*PTN* physical training and nutritional intervention group, *SOSU* social support group, *SPPB* Short Physical Performance Battery, *PASE* Physical Activity Scale for the Elderly, *MNA®-LF* Mini Nutritional Assessment Long-Form

*P < 0.05 for within-group change score from baseline to follow-up; **P < 0.001 for within-group change score from baseline to follow-up analysed with a Paired t-test

^a^ß is unstandardised regression coefficient; ^b^Analyses of covariance (ANCOVA) were used to examine differences between groups adjusted for baseline measure, sex and age, with the group as the fixed between-subjects factor

Table 3 shows which changes in baseline variables (maximum handgrip strength, SPPB score, PASE score, MNA®-LF score) and group were associated with changes in several QoL domains (overall QoL, social relationship, social participation, past, present and future activities). In this regard, changes in maximum handgrip strength and physical function were found to be significantly related to changes in overall QoL. Changes in maximum handgrip strength and positive changes in physical activity were significantly related to changes in social relationship. Further, changes in nutritional status and physical activity were found to be related to social participation. Finally, changes in physical function and participants who were allocated to the PTN group were found to be significantly related to changes in the past, present and future activities domain. Interestingly we found a negative association between changes in nutritional status and changes in the past, present and future activities domain.

**Table 3:** Changes in independent variables explaining changes in several QoL domains

Variables	Change in overall QoL^a^	Change in social relationship^a^	Change in social participation^a^	Change in past, present and future activities^a^
	Standardised ß (95% CI)	Standardised ß (95% CI)	Standardised ß (95% CI)	Standardised ß (95% CI)
PTN group	0.10 (−2.90 to 8.58)	−0.04 (−9.41 to 6.96)	0.01 (−5.13 to 5.63)	0.28 (0.88 to 7.95)^*^
Maximum handgrip strength^b^	0.38 (0.48 to 1.80)^**^	0.29 (0.19 to 2.01)^*^	−0.02 (−0.65 to 0.58)	0.13 (−0.17 to 0.63)
SPPB score^b^	0.23 (0.59 to 2.35)^*^	0.03 (−1.82 to 2.23)	−0.81 (−1.84 to 0.92)	0.25 (0.27 to 1.58)^*^
PASE score^b^	0.05 (−0.08 to 0.15)	0.31 (0.04 to 0.38)^*^	0.19 (−0.02 to 0.20)^*^	−0.12 (−0.12 to 0.04)
MNA®-LF score^b^	0.13 (−0.03 to 1.36)	−0.04(−1.5 to 0.79)	0.32 (0.26 to 1.82)*	−0.29 (−1.19 to −0.17)*

*Note*: Multiple linear regression analyses adjusted for sex and age; *PTN* physical training and nutritional intervention, *SPPB* Short Physical Performance Battery, *PASE* Physical Activity Scale for the Elderly, *MNA®-LF* Mini Nutritional Assessment Long-Form, *95% CI* 95% confidence interval

*P < 0.05; **P < 0.001

^a^Dependent variables (differences of post-test minus pre-test); ^b^Independent variables were mean changes in baseline variables (differences of post-test scores minus pre-test scores)

## Discussion

The combined nutrition and exercise intervention did not lead to significant improvements in QoL compared to social support only. However, even though we only found significant group differences in the past, present and future activities, it is important to note that we saw some evidence of greater within group improvements in the PTN group, particularly in overall QoL, social relations and social participation. The positive effects with regard in the past, present and future activities domain, it can be assumed that the activities undertaken in the home visits (e.g., conversations about nutrition, exercise and social support), as a result of the intervention programme, was perceived as positive, and that this current status should be upheld in order to live independently at home as long as possible. In this context, the study of Salkeld et al. [44] showed that, after a hip fracture, 80% of older women stated that they preferred death to losing their independence or to possible admission to a nursing home.

Although the physical performance and physical activity in our study shows a significant improvement, there were no significant changes in the physical health domain. It can be expected that an active lifestyle preserves physical function in older adults [45], which in turn leads to higher levels of QOL scores in domains related to physical health [46]. The present study was not able to demonstrate greater values in the physical health domain. One possible explanation for the unchanged values is that almost 68% of the participants had at baseline a low performance score (less than 6 points), 24% had a moderate score and 8% had a high score (more than 10 points) in the SPPB. Thus, a total score lower than 6 points is considered an indication of serious functional limitations in older adults [47]. These results imply that the physical health (e.g. pain, discomfort, sufficient sleep, rest and good work capacity) due to serious functional limitations in this population may be responsible for the lack of change. However, as evident in our study the SPPB score improved of at least 1 point. An improvement of one to two points is already substantial [48–50]. Thus, this trial is likely to be worthwhile considering the importance of physical activity in homebound older people.

A further explanation for unchanged value can be partly explained by the fact that home visits with social support alone had a tendency towards improvement and due to the limited statistical power. A study in South Korea indicated that social support influences QoL in older adults in a positive way [51]. Moreover, a two-year longitudinal study showed that social frailty (i.e., living alone, lack of social contacts and lack of social support) was associated with receiving nursing care and the number of contacts with health care professionals [52]. It is not surprising that in the qualitative study of Puts et al. [53], social contact was identified as the most important factor for pre-frail or frail people. In addition, the results of Seeman [54] demonstrated an association between increased levels of social support and reduced risk of physical disease, mental illness and mortality, while, in contrast, older participants who were lacking in social relationships and contacts were found to have a higher possibility of suffering from loneliness during their daily activities [55]. Hence, it is possible that a supportive atmosphere with social exchange, as experienced by the SOSU group, might be positively linked to the minor changes in the QoL domains. However, surprisingly, the SOSU group showed no significant change in any QoL domain. One possible explanation for this could be that the highly motivated participants recruited through the media, who were allocated to the SOSU group, were dissatisfied with the lack of activities or, as can be seen in Fig. 1, were disappointed not to be in the PTN group.

The adherence rate of 75% in our study, which is comparable to similar studies analysed in the meta-analysis of Chou et al. [56], was essential to the success of our intervention, and likely resulted from the social interaction and the encouragement of the volunteers. In addition, the home-based exercises were not connected to any adverse events (apart from one person with back pain), and appear to be safe. In summary, this highlights the importance of the measures being tailored to the resources of the pre-frail and frail older adults, in order to achieve a high level of adherence and, thereby, an effect.

There is large body of evidence supports the claim that physical exercise has a positive impact on heath related QoL, even if it is not performed simultaneously to a nutritional intervention [57, 58]. As presented in Table 3, changes in nutritional status were found to be related to changes in social participation. In this respect, the study of Kwon et al., [59] who demonstrated that an exercise programme alone did not result in a significant change in health-related QoL when compared to a combined exercise and nutritional intervention programme. However, interestingly we found a negative association between change in nutritional status and change in the past, present and future activities domain when adjusted for sex and age, which is difficult to interpret.

Finally, in the context of the linkage between older lay volunteers and pre-frail or frail older adults, Puts et al. [53] stated that, for non-frail people, health plays a central role for a good QoL, and that the importance of helping others, as well as social activity, is also beneficial for a good QoL. Considering that many health professionals have limited time resources [60] to deliver exercise interventions during a routine home visit, social interchange is understandably not always possible. Thus, this kind of association can yield double benefits. On the one hand, the health status of both the pre-frail and frail subjects and the older lay volunteers can improve, due to the active engagement with nutritious food and physical training and, in particular, through the health education. However, the improvement in the health status of the volunteers still needs to be examined. While using senior volunteers in health promotion has many benefits [61] (e.g. cost effective, feeling useful and self-fulfilled, be part of a community, reduced symptoms of depression) there are also some disadvantages. For instance, the burden on volunteers in respect to training and service delivery can be overwhelming, and due to disability and illness, which are more prevalent in older age groups, the dropout rate can be high [62]. However, the pairing between lay volunteers and frail older adults can work well, provided that lay volunteers are well trained to ensure that they do not operate outside their sphere of competence.

The mayor strength of this study is the implementation of the lay-led home-based programme, which adds new information to the knowledge about the effectiveness of nutritional education, social support and physical training delivered by lay volunteers. Such “buddy system” could be used as a resource in terms of reaching frail older adults who are bound to their own homes.

### Study limitations

The study does have some limitations. Firstly, there was no inactive control group. Since nonspecific input factors such as social support can improve QoL [63], it is difficult to attribute the changes to the specifics of the home-based programme. However, the aim of this randomised controlled trial was to examine if the approach is feasible, and whether additional nutritional and physical training intervention is more effective than social support alone. Secondly, a large proportion of the study participants (95%) were enrolled via the media due to the lack of success in the hospital environment. It cannot be ruled out that a sample of well-motivated participants and only those interested participated in the study. Thus, the participants were largely self-selected. Therefore, this might not only limit the generalizability of the findings, but also result in failure to target those who may most need and benefit from such an intervention. Thirdly, the sample size of 80 subjects, which is of small to medium scope, is a major limitation of the study, especially for the sub-group analyses and the more complex statistical procedures. However, the sample size was calculated based on the data of the pre-study [29]. Finally, blinding of participants and researchers was not possible, due to the nature of the exercise-based intervention. Thus, the possibility of observer bias must be taken into account.

## Conclusion

The linking of lay volunteers with frail older adults in a structured programme with physical training and nutritional intervention was not effective compared to social support on several QoL domains in community-dwelling pre-frail and frail older adults. However, the small but significant improvement within the PTN group suggests that a home-based physical and nutritional intervention delivered by volunteers may influence the QoL in a positive way.

## Abbreviations

ANCOVA: Analysis of covariance
MMSE: Mini Mental State Examination (MMSE)
MNA-SF/LF: Mini Nutritional Assessment Short-Form/Long-Form
OR: Odds ratio
PASE: Physical Activity Scale for the Elderly
PTN: Physical training and nutritional intervention group
QoL: Quality of life
SD: Standard deviation
SHARE-FI: Frailty Instrument for Primary Care of the Survey of Health, Ageing and Retirement in Europe
SOSU: Social support group
SPPB: The Short Physical Performance Battery
WHOQOL: The World Health Organization Quality of Life
